# Population data science

**DOI:** 10.1590/S2237-96222022000300001

**Published:** 2022-12-19

**Affiliations:** 1 Universidade Federal do Rio de Janeiro, Rio de Janeiro, RJ, Brazil

The rapid evolution of information technology provides unprecedented growth in data production, storage, exchange, processing and analysis capacity.^1^ In addition to structured data, various unstructured data formats are currently made available. Structured data are used in administrative databases, which are produced and used in typical Public Health activities. Regarding this type of database, each row corresponds to an entity, usually an individual, and each column corresponds to an attribute of that entity (for example, date of birth). In turn, unstructured data present different formats, which include, for example, texts in documents or social networks, images, and sensor outputs. These data, especially when linked, have been increasingly used in health, research, surveillance and evaluation activities, as well as in decision-making.^2^

This new complex data ecosystem encouraged researchers from the International Population Data Linkage Network (IPDLN; available at http://www.ipdln.org/) to publish an article in 2018 proposing the creation of a new disciplinary field, which they called population data science.^3^ Population Data Science is “a multi-disciplinary field aimed at obtaining population-level insights with public value by organizing, linking or otherwise integrating and analyzing data that pertain to individuals and their social, economic, biological and environmental characteristics and contexts.” Or, in short, “the science of data about people”.^3^ The intensive use of complex databases – resulting from the linkage or integration of individual data of a diverse nature, with population coverage, for the generation of evidence of value to society – is the main characteristic of this new disciplinary field, and that distinguishes it from other related disciplines, such as data science and informatics.^3^

With regard to this disciplinary field, two important challenges must be addressed. Initially, it is necessary to implement technical infrastructure and data access governance policies that respect ethical and privacy norms, as well as meet society's expectations.^4^ The data center model – which operates as a reliable third party in the relationship between database custodians and researchers and other actors interested in the use of linked databases – was adopted by several countries,^5^ ensuring the balance between guaranteeing privacy preservation and enabling efficient and secure access to linked data, through projects that aim to produce knowledge that is relevant to society.^4^

The second challenge is related to the lack of familiarity of researchers and managers with the use of large and complex secondary databases for surveillance, evaluation and research purposes, which may lead to misinterpretation of the evidence generated.^2,6^ Unlike primary data collected to answer a specific research question, researchers and managers generally have no control over the generation and processing of secondary datasets. When using a linked secondary database, it is necessary to know several aspects, including population coverage, field completeness, presence of duplicate records, proportion of missing data, reliability and validity of data, the attribute coding systems, the algorithms used for data transformation, and the data linkage process, including linking errors.^2^ Additionally, taking into consideration that these data are collected in different locations and over long periods of time, it is important to evaluate whether these aspects are stable over time, and whether there are regional inequalities.

In Brazil, we have a consolidated experience in the availability and use of unidentified individual microdata for research, evaluation and surveillance purposes. However, in order for this successful experience to advance, several actions would be necessary, including the implementation of technical infrastructure and governance policies for access to linked unidentified microdata, the dissemination and updating of the metadata of the original databases and derived linked datasets, and the training of researchers and managers in the domains that comprise the field of population data science.
